# Determinants of depression, problem behavior, and cognitive level of adolescents in China: Findings from a national, population-based cross-sectional study

**DOI:** 10.3389/fpsyt.2023.1159739

**Published:** 2023-04-06

**Authors:** Yusang Dong, Xinyu He, Lizhen Ye, Lidan Sun, Jiabin Li, Jingfang Xu, Yuechong Cui, Ziqiao Li, Lidan Hu, Guannan Bai

**Affiliations:** ^1^Children’s Hospital, Zhejiang University School of Medicine, National Clinical Research Center for Child Health, Hangzhou, Zhejiang, China; ^2^Department of Child Health Care, Zhejiang University School of Medicine, National Clinical Research Center for Child Health, Children’s Hospital, Hangzhou, Zhejiang, China; ^3^Department of Public Health, Erasmus University Medical Center, Rotterdam, Netherlands; ^4^Department of Pharmacy, Zhejiang University School of Medicine, National Clinical Research Center for Child Health, Children’s Hospital, Hangzhou, Zhejiang, China; ^5^Department of Orthopaedics, Zhejiang University School of Medicine, National Clinical Research Center for Child Health, Children’s Hospital, Hangzhou, Zhejiang, China; ^6^Yiwu Maternity and Children Hospital, Yiwu Branch of Children’s Hospital Zhejiang University School of Medicine, Yiwu, Zhejiang, China

**Keywords:** adolescent, depression, problem behavior, cognitive development, cross-sectional study

## Abstract

**Introduction:**

We aimed to assess the associated factors for adolescent depression, problem behavior and cognitive level in China.

**Methods:**

A total of 2,584 adolescents aged from 10 to 15 years old in 2018 were included for analyses. Information on a comprehensive set of potential determinants was collected by the questionnaire, including demographic, health-, school- and family-related factors. Differences in average scores of depression, problem behavior, and cognitive level across subgroups were assessed by two independent sample *t*-tests and one-way analysis of variance (ANOVA). The clinical relevance among subgroups was assessed by the effect size. Multivariate linear regression models were applied to identify the statistically significant determinants.

**Results:**

School-related factors and parental depressive status were strongly associated with depression. Low maternal education, poor/bad health of adolescents, high academic pressure, and parental depression were significantly associated with behavior problems. The socioeconomic factors, poor academic performance and father’s depression were significantly associated with adolescent cognitive level.

**Discussion:**

Multiple associated factors were identified for depression, problem behavior, and cognition of Chinese adolescents, which will provide insights into developing more targeted public health policies and interventions to improve their mental health.

## 1. Introduction

Adolescence is an essential period of transition from childhood to adulthood with dramatic changes in physical and mental well-being as well as social functioning. In particular, adolescents are at relatively high risk of mental health disorders, and behavioral and cognitive problems that affect their long-term health and limit their life to their full potential. Mental health disorders affect 10–20% of children and adolescents worldwide, and the incidences are rising substantially, which remains a challenging public health issue (1–3). For instance, unipolar depressive disorder is common in adolescents worldwide, which is a major risk factor for suicide and a leading cause of early death (4). The prevalence of depression among adolescents was 24.6% in China, 36.0% in Australia, 40.1% in western Europe, and 49.0% in North America (5, 6). During adolescence, individuals are sensitive to certain behavior disorders, such as attention-deficit/hyperactivity disorder, oppositional defiant disorder, and conduct disorder (7–9). In addition, adolescence is also an important period for cognitive development, including language development, reasoning skills, critical thinking, and abstract thinking, which will influence academic performance at school and career achievement at work in the future.

In the current study, we focused on the associated factors of mental health, behavior, and cognition in Chinese adolescents. As for individual factors, the anthropometric status, such as overweight and obesity, increased the risk of depression among adolescents (10). The CFPS data showed that the cognitive level was related to key demographic and social characteristics, such as age, gender, education, and residence (11). The depressed adolescents had fewer friends and were less popular. They more often had suicidal thoughts (12). Family-related factors such as marital status, parenting style, parents’ mental health and family function may impact adolescents’ psychological status (13). Parental depression during adolescence was associated with adverse offspring cognitive development (14). Adolescents growing up in poverty were associated with their mental health and cognition (15, 16). The school environment plays an essential role in terms of student development. For instance, school violence, being unhappy with school performance, feeling unsafe at school, self-perception regarding being overweight, and being female those factors were significantly related to mental health difficulties of adolescents (17, 18). Therefore, academic pressure and peer pressure significantly affect adolescents’ mental health (19). Additionally, favorable socioeconomic status, resources, culture, and policy were also reported by the previous studies as protective factors for adolescents’ mental and cognitive development (20).

In 2020, the Chinese Education Ministry published a policy by including depression screening in regular health check-ups at school. Adolescents’ mental health, problem behavior, and cognitive development may share common determinants, including individual, family, school, and social-related factors (21). Most previous studies focused on a single outcome and a limited amount of potential determinant variables. CFPS measured many variables in a large sample of Chinese children and their families, which made it feasible to assess a comprehensive set of potential determinant factors for adolescents’ depression, behavior problems, and cognitive level. Therefore, we aimed to identify these determinant factors, especially the modifiable ones, which helped to make evidence-based policy and eventually to improve their mental health and cognition.

## 2. Materials and methods

### 2.1. Data sources

The present study was embedded in the China Family Panel Studies (CFPS), and detailed information has been described elsewhere (22). The access link is http://www.isss.pku.edu.cn/cfps/en/. CFPS is a nationwide, longitudinal survey launched in 2010 by the Institute of Social Science Survey (ISSS) of Peking University, China. The sample was selected from 25 provinces/municipalities/autonomous regions, with a targeted sample size of 16,000 households (23). Subjects in the survey were all family members in the same households, representing 95% of the Chinese population. The data were collected every 2 years. It aims to collect communities, families, and individual-level data in contemporary China which can reflect the changes in society, economy, education, and health. The baseline data was collected in 2010 through a multi-stage stratified sampling approach (24, 25). In the present study, we used the survey data that were collected in 2018. The study was conducted according to the Declaration of Helsinki (26) and was approved by the Ethics Committees of the Institution of Social Science Survey, Peking University (the approval number: IRB00001052–14010).

### 2.2. Study population

**FIGURE 1:**
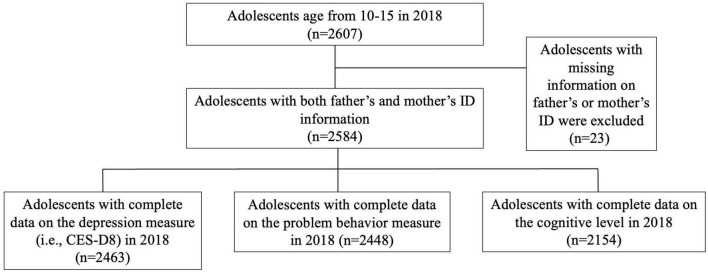
Flow chart of the study population.

Figure 1 shows the flow chart of selecting the study population. First, we selected adolescents aged 10–15 years who participated in the CFPS survey in 2018 and who were capable to fill in the self-reported questionnaires (*n* = 2,607) (27). Second, we excluded adolescents whose parents did not have identification information (*n* = 23). Third, we excluded adolescents without information on measurements of depression (*n* = 121), problem behavior (*n* = 136), and cognitive level (*n* = 430), respectively. Thus, there were three datasets for the final analysis (depression: *n* = 2,463; problem behavior, *n* = 2,448; and cognitive level, *n* = 2,154).

### 2.3. Measurements of outcomes and determinants

#### 2.3.1. Measurement of depression

The CFPS applied a special version of the eight-item Center for Epidemiological Studies Depression Scale (CES-D8), which is based on a widely-used instrument to measure depression symptoms (28). Instead of using 20 questionnaires, CFPS used eight questions according to the Health and Retirement Study (HRS) including the self-reported frequency of depression feelings and relevant behaviors in the past week. The eight questions items were as below: (1). I felt depressed; (2). I felt that everything I did was an effort; (3). My sleep was restless; (4). I was happy. (5). I felt lonely; (6). I enjoyed life; (7). I felt sad; (8). I could not get “gonging”. There were four options, i.e., (1) rarely (less than 1 day), (2) sometimes or occasionally (1–2 days), (3) often (3–4 days), (4) most of the time or always (5–7 days). The scores of all items were summed up that ranges from 8 to 32. A higher score indicates a higher level of depression. CES-D has been previously validated in the Chinese population with good reliability and validity (29, 30). The Cronbach’s alpha of CES-D was 0.616 in the present study.

#### 2.3.2. Measurement of problem behavior

Problem behavior for adolescent are those that aren’t considered typically acceptable, and Jessor described problem behavior as any behavior that deviates from both social and legal norms (31, 32). It includes internalizing problem behavior and externalizing problem behavior. CFPS measured adolescents’ problem behavior in 2018 for the first time a self-reported and brief instrument that was developed in accordance with the Early Childhood Longitudinal Study. This brief version contains eight questions for measuring the internalizing problem behavior and six for the externalizing problem behavior. It has acceptable reliability with Cronbach’s alpha coefficient of the internalizing problem dimension as 0.65, and 0.64 for the externalizing problem dimension. The CFPS recommended researchers calculate dimension scores (i.e., the score of the internalizing problem behavior and the score of externalizing problem behavior) and the total score (27). In this study, we have internalizing problem score, externalizing problem score, and total score by adding two scores together. A higher score indicates more severe problem behavior.

#### 2.3.3. Measurement of cognitive level

The cognition is defined as the combination of “crystallized intelligence” and “fluid intelligence” (11). The “crystallized intelligence” is referred to as acquired knowledge through learning, experience, and education. The “fluid intelligence” indicated the ability to reason abstractly and to solve problems (33). In the CFPS, the adolescent cognitive level was measured by a set of words and math questions. The word test consists of eight groups of questions, and each group contains 34 words from easy to difficult ones. The math test contains four groups of questions including addition, subtraction, multiplication, division, exponent, logarithm, trigonometric function, number sequence, permutation, and combination (26). Both word and math tests have been widely adopted and validated in previous studies to measure cognitive ability among adolescents and adults in the Chinese population (34, 35). We combine word and math tests into total scores of a word and math test by adding two test scores together. During the test, questions were randomly selected by the computer, and the difficulty of each group of questions was equal. A higher score indicates a higher cognitive level (36). The Cronbach’s alpha was 0.756 in our study.

#### 2.3.4. Measurements of potential determinants

##### 2.3.4.1. Demographic characteristics

Information on age, gender, birth order, mother’s educational level, annual household income, and resident location were collected by the question in 2018. The birth order was divided into three groups, i.e., first child, second child, third or more child. Mothers’ education levels were categorized into three groups, i.e., primary school or low, junior or secondary school, and high school or above. The annual household income was divided into quartiles. The residence was categorized as “rural and urban areas.”

##### 2.3.4.2. Adolescents’ health-related factors

Information on health-related factors includes Body Mass Index (BMI), sleeping duration, and self-rated health status. BMI was calculated by dividing weight (kilogram) by the square of height (meter) and then divided into four subgroups, i.e., underweight, normal, overweight, and obesity according to the World Health Organization (WHO) table of BMI-for-age of boys and girls aged from 5 to 19 years old. Adolescents are recommended to have 8 h of sleeping time at night which refers to “appropriate sleeping time” (37). We accordingly divided the sleep duration into two groups, i.e., <8 h per day, and ≥ 8 h per day. Self-rated health status was measured by one question, i.e., “How would you rate your health status in the past two weeks?” There were five options, i.e., Excellent, good, fair, bad, and very bad.

##### 2.3.4.3. Adolescents’ school-related characteristics

Class ranking referred to the results of the lasted examination, and CFPS study was categorized into a 0–10th percentile, 11th–25th percentile, 26th–50th percentile, 51th–75th percentile and 76th–100th percentile. We combine middle class ranking as an 11th–75th percentile, and have “high (the first ten percentile)”, “middle (11th to 75th percentile)”, and “last (76th to 100th percentile)” in three groups. Academic pressure was measured by one self-rated question with three options (i.e., low, moderate, and high academic pressure). Popularity was measured by a 0–10 scale and we used the median as a cutoff to divide the score into two groups, the advantage of using the median is that it is very stable and less susceptible to outliers. Thus, we divided the popularity category into two parts, being less popular when the score was less or equal to 7 and being popular when the score was greater than 7.

##### 2.3.4.4. Parental depression

We measured the depression of mothers and fathers using the CES-D8 instrument. CFPS 2018 used the Equi-percentile equating method to equalize the scores of the CES-D8 and CES-D20 sets of items. We then chose the 28 points as the cutoff and divided parents’ depression into two groups which were depression and non-depression.

### 2.4. Statistical analyses

We first conducted descriptive analyses to characterize the study population (*n* = 2584). Secondly, we assessed the differences in average scores of adolescent depression, problem behavior, and cognitive level across subgroups of determinant variables by two independent sample *t*-tests for the comparison between two subgroups and one-way analysis of variance (ANOVA) for the comparison among three or more subgroups. The clinical relevance as indicated by group differences was assessed by effect size. We used Cohen’s d for the independent *t*-tests and partial eta square ($\eta_{p}^{2}$) for ANOVA. Based on the Benchmarks suggested by Cohen, effect sizes are indicated as small (0.2 ≤ d < 0.5, 0.01 ≤ $\eta_{p}^{2}$ < 0.06), medium (0.2 ≤ d < 0.5, 0.06 ≤ $\eta_{p}^{2}$ 0.14), and large (d ≥ 0.8, $\eta_{p}^{2}$ ≥ 0.14) (38). Regarding the missing data, we conducted multiple imputations that were based on the relationships between all variables. Five imputed datasets were generated. We used the multivariate linear regression models to assess associations between the potential determinant variables and the outcomes, i.e., depression, problem behavior, and cognitive level. In addition, we conducted multivariate linear regression analyses again in the non-imputed data.

All analyses were conducted in SPSS25.0 (IBM Corp., Chicago, U.S.). *P* < 0.05 indicates a statistical significance.

## 3. Results

### 3.1. General characteristics of the study population

Table 1 shows the general characteristics of the study population. The average age was 12.4 (SD: 1.7) years. 52.8% were boys. 57.6% of adolescents were the first child in the family. 54.5 % of families live the rural area. 11% of mothers had high school or higher degrees. 15.8% of adolescents were overweight or obese. 74.8% of adolescents had 8 h or longer time of sleeping. 33.3% reported their health status as “excellent” while 4.5% as “very bad”. 5.8% had a high ranking (i.e., top 15 percentile) in class while 20.1% had a low ranking (i.e., 0–10th percentile). 26.9% reported a high level of academic pressure; 48.5% rated themselves as less popular at school. 18.9% of mothers and 14.1% of fathers had depression as indicated by the CES-D8.

**TABLE 1:** General characteristics of the study population (*n* = 2,584).

Characteristics	Values (percentage)
**Demographic characteristics**	
**Age, *n* (%)**	
10	454 (17.6)
11	440 (17.0)
12	427 (16.5)
13	456 (17.6)
14	450 (17.4)
15	357 (13.8)
Mean (SD)	12.4 (1.7)
**Gender, *n* (%)**	
Boys	1,364 (52.8)
Girls	1,220 (47.2)
**Birth Order, *n* (%)**	
First	1,488 (57.6)
Second	896 (34.7)
Third or more	200 (7.7)
**Mother’s educational level, *n* (%)**	
High school or higher	290 (11.2)
Secondary school	600 (23.2)
Primary school or less	841 (32.5)
Missing	853 (33.0)
**Household income per year, *n* (%)**	
1st quartile	601 (23.3)
2nd quartile	603 (23.3)
3rd quartile	639 (24.7)
4th quartile	553 (21.4)
Missing	188 (7.3)
**Residence, *n* (%)**	
Rural	1,408 (54.5)
Urban	1,057 (40.9)
Missing	119 (4.6)
**Adolescents’ health-related factors**	
**Body mass index, *n* (%)**	
Underweight	739 (28.6)
Normal	1,231 (47.6)
Overweight/obesity	407 (15.8)
Missing	207 (8.0)
**Sleeping duration, n (%)**	
<8 h	517 (20.0)
≥8 h	1,932 (74.8)
Missing	135 (5.2)
**Self-rated health status, *n* (%)**	
Excellent	861 (33.3)
Good	862 (33.4)
Fair	740 (28.6)
Poor and very bad	115 (4.5)
Missing	6 (0.2)
**School-related factors**	
**Class ranking, *n* (%)**	
0-10th percentile	520 (20.1)
11-75th percentile	1,314 (50.9)
76th-100th percentile	151 (5.8)
Schools do not publish ranking or missing	599 (23.2)
**Academic pressure, *n* (%)**	
Low	844 (32.7)
Moderate	907 (35.1)
High	695 (26.9)
Missing	138 (5.3)
**Self-rating popularity, *n* (%)**	
Less popular (≤7)	1,254 (48.5)
Popular (>7)	1,216 (47.1)
Missing	114 (4.40)
**Parental mental health indicators**	
**Father’s depression test, *n* (%)**	
Non-depression	1,508 (58.4)
Depression	365 (14.1)
Missing	711 (27.5)
**Mother’s depression test, *n* (%)**	
Non-depression	1,519 (58.8)
Depression	489 (18.9)
Missing	578 (22.3)
**Outcome measures (adolescents)**	
Depression test, mean (SD)	11.91 (3.2)
Missing, *n* (%)	121 (4.7)
**Problem behavior**	
Internalizing problem test, mean (SD)	17.3 (4.8)
Externalizing problem test, mean (SD)	10.0 (3.4)
Total scores, mean (SD)	27.3 (7.1)
Missing, *n* (%)	136 (5.3)
**Cognitive level**	
Word test mean (SD)	23.8 (5.9)
Math test, mean (SD)	11.6 (3.9)
Total scores, mean (SD)	35.4 (8.9)
Missing, *n* (%)	478 (18.5)

Values in this table are numbers, percentages, means, and standard deviations (SD).

### 3.2. Differences in the mean scores of outcome measures across subgroups

Table 2 presents the difference in the mean scores of outcome measures across subgroups as well as the values of effect sizes. The average score of adolescent depression was statistically different across subgroups of birth order, maternal education level, BMI, sleeping duration, self-rated health status, class rank, academic pressure, self-rating popularity, father’s depression, and mother’s depression status (*p*-values < 0.05). The average score on the problem behavior test was statistically different across the subgroups of age, mother’s educational level, household income, residence area, self-rated health status, class rank, academic pressure, self-rated popularity, father’s depression, and mother’s depression status (*p*-values < 0.05). Regarding the internalizing and externalizing problem behavior, the pattern between subgroups was similar to that of the total score. The average score on the cognitive test was statistically different across subgroups of age, gender, birth order, mother’s educational level, household income, residence area, BMI, sleep duration, self-rated health status, class rank, academic pressure, and father’s depression (*p*-values < 0.05). According to the word and math tests, there was a slight difference in the pattern of the total score. In addition, results of effect size show that the large difference in the average scores of word test, match test, and total cognitive level test existed across subgroups of age ($\eta_{\text{p}}^{2}$ 0.243, $\eta_{\text{p}}^{2}$ 0.352, and $\eta_{\text{p}}^{2}$ 0.349, respectively).

**TABLE 2:** Differences in the average scores of depression test (*n* = 2,463), problem behavior test (*n* = 2,448), cognitive level test(*n* = 2,106).

	Depression (*n* = 2463)		Problem behavior(*n* = 2448)						Cognitive level test(*n* = 2106)					
**Characteristics**			**Internalizing problem behavior**		**Externalizing problem behavior**		**Total score**		**Word test score**		**Math test score**		**Total score**	
	**MD (SD)**	**Effect size**	**MD (SD)**	**Effect size**	**MD (SD)**	**Effect size**	**MD (SD)**	**Effect size**	**MD (SD)**	**Effect size**	**MD (SD)**	**Effect size**	**MD (SD)**	**Effect size**
**Demographic characteristic**														
Age, *n* (%)		0.01	***	0.01		0.01	*	0.01	***	0.24	***	0.35	***	0.35
10	11.92 (3.37)		17.19 (5.00)		10.31 (3.57)		27.50 (7.50)		19.13 (5.91)		8.05 (2.22)		27.18 (7.08)	
11	11.54 (3.12)		16.78 (4.76)		10.05 (3.39)		26.82 (7.25)		21.23 (5.97)		9.52 (2.67)		30.75 (7.66)	
12	11.79 (2.82)		16.77 (4.91)		9.57 (3.38)		26.34 (7.30)		24.05 (4.90)		11.44 (2.84)		35.48 (6.83)	
13	12.08 (3.50)		17.82 (4.75)		10.07 (3.46)		27.88 (7.11)		25.09 (5.14)		12.63 (3.17)		37.71 (7.07)	
14	12.02 (2.96)		17.48 (4.55)		9.90 (3.20)		27.37 (6.72)		26.57 (4.52)		13.61 (3.79)		40.18 (7.27)	
15	12.16 (2.97)		17.84 (4.60)		10.04 (2.96)		27.88 (6.50)		27.62 (4.14)		14.96 (4.05)		42.58 (7.05)	
Gender, *n* (%)		0.07	*	0.08	***	0.18		0.06	***	0.14		0.04	**	0.11
Boys	11.80 (3.03)		17.11 (4.78)		10.38 (4.50)		27.49 (7.20)		23.39 (6.18)		11.51 (3.92)		34.90 (9.15)	
Girls	12.04 (3.27)		17.50 (4.79)		9.56 (3.13)		27.06 (7.00)		24.26 (5.64)		11.66 (3.90)		35.92 (8.54)	
Birth Order, *n* (%)	*	0.01		0.01	*	0.01		0.01	***	0.01	**	0.01	***	0.01
First	11.89 (3.23)		17.30 (4.76)		9.89 (3.25)		27.19 (6.96)		24.31 (5.76)		11.83 (4.00)		36.14 (8.82)	
Second	11.82 (3.02)		17.17 (4.91)		10.01 (3.43)		27.19 (7.33)		23.31 (5.96)		11.34 (3.76)		34.65 (8.70)	
Third or more	12.50 (3.05)		17.83 (4.40)		10.61 (3.72)		28.45 (7.12)		22.43 (6.73)		10.91 (3.80)		33.34 (9.56)	
Mother’s educational level, *n* (%)	***	0.01	***	0.02	***	0.03	***	0.03	***	0.02	***	0.03	***	0.03
High school or higher	11.10 (2.63)		16.10 (4.36)		8.99 (2.71)		25.08 (6.08)		25.70 (4.77)		13.03 (3.70)		38.73 (7.63)	
Secondary school	11.62 (3.18)		16.68 (4.66)		9.43 (3.10)		26.11 (6.79)		24.40 (5.47)		12.13 (3.74)		36.53 (8.39)	
Primary school or less	12.04 (3.12)		17.63 (4.90)		10.42 (3.57)		28.05 (7.45)		23.30 (6.08)		11.29 (3.90)		34.59 (8.91)	
Household income per year, *n* (%)		0.01	*	0.01	**	0.05	**	0.01	***	0.01	***	0.03	***	0.02
1st quartile	12.14 (3.12)		17.72 (4.76)		10.19 (3.50)		27.91 (7.20)		23.30 (5.90)		10.96 (3.91)		34.25 (8.73)	
2nd quartile	11.97 (3.23)		17.44 (4.85)		10.15 (3.31)		27.59 (7.19)		24.03 (5.90)		11.68 (3.83)		35.71 (8.69)	
3rdquartile	11.78 (3.18)		16.91 (4.71)		9.90 (3.39)		26.81 (7.11)		23.91 (5.90)		11.66 (3.82)		35.57 (8.84)	
4th quartile	11.62 (2.98)		17.06 (4.72)		9.59 (3.00)		26.65 (6.61)		25.02 (5.28)		12.77 (3.81)		37.79 (8.19)	
Residence, *n* (%)		0.07		0.08	***	0.18	**	0.14	***	0.30	***	0.32	***	0.34
Rural	12.00 (3.09)		17.47 (4.79)		10.25 (3.43)		27.72 (7.12)		23.01 (6.29)		11.11 (3.88)		34.11 (9.16)	
Urban	11.77 (3.21)		17.10 (4.77)		9.64 (3.23)		26.74 (7.06)		24.89 (5.27)		12.35 (3.81)		37.24 (8.16)	
**Adolescents’ health-related factors**														
Body mass index, *n* (%)	*	0.01		0.01		0.01		0.01	***	0.01	***	0.01	***	0.01
Underweight	12.02 (2.98)		17.35 (4.80)		10.02 (3.47)		27.37 (7.22)		23.90 (5.58)		11.46 (3.80)		35.36 (8.32)	
Normal	11.89 (3.19)		17.28 (4.70)		9.85 (3.25)		27.13 (6.94)		24.39 (5.79)		12.01 (3.97)		36.40 (8.82)	
Overweight/obesity	11.54 (3.02)		17.26 (5.20)		10.32 (3.60)		27.58 (7.73)		22.71 (6.41)		10.95 (3.76)		33.66 (9.17)	
Sleeping duration, *n* (%)	***	0.16		0.06		0.08		0.01	***	0.38	***	0.40	***	0.45
<8 h	12.31 (3.17)		17.51 (4.66)		9.78 (3.13)		27.29 (6.65)		25.60 (5.40)		12.91 (4.17)		38.51 (8.70)	
≥8 h	11.79 (3.13)		17.24 (4.82)		10.04 (3.41)		27.28 (7.23)		23.35 (5.98)		11.25 (3.77)		34.60 (8.74)	
Self-rated health status, *n* (%)	***	0.04	***	0.01	***	0.02	***	0.02	***	0.01	***	0.01	***	0.01
Excellent	11.26 (2.95)		16.67 (4.90)		9.72 (3.47)		26.39 (7.38)		23.44 (6.02)		11.27 (3.81)		34.72 (8.77)	
Good	11.74 (2.91)		17.21 (4.57)		9.75 (3.16)		26.96 (6.75)		24.19 (5.90)		12.05 (3.90)		36.25 (8.93)	
Fair	12.60 (3.34)		17.95 (4.83)		10.39 (3.30)		28.35 (7.02)		24.08 (5.70)		11.49 (3.93)		35.56 (8.61)	
Poor and very bad	13.70 (3.45)		18.52 (4.57)		11.43 (3.74)		29.95 (7.06)		21.51 (6.70)		10.71 (4.25)		32.22 (10.02)	
**School-related factors**														
Class ranking, *n* (%)	***	0.01	***	0.01	***	0.03	***	0.02	***	0.04	***	0.03	***	0.05
0-10th percentile	11.50 (2.88)		16.71 (4.28)		9.17 (3.04)		25.88 (6.29)		25.11 (5.34)		12.38 (3.86)		37.49 (8.36)	
11-75th percentile	11.94 (3.11)		17.48 (4.89)		10.12 (3.31)		27.60 (7.21)		24.37 (5.65)		12.08 (3.91)		36.45 (8.64)	
76th-100th percentile	12.95 (3.24)		17.95 (5.02)		11.58 (3.73)		29.52 (7.42)		20.25 (7.61)		9.53 (4.05)		29.78 (9.96)	
Academic pressure, *n* (%)	***	0.03	***	0.05	***	0.02	***	0.05	**	0.01	***	0.01	***	0.01
Low	11.39 (3.03)		16.11 (4.75)		9.54 (3.31)		25.65 (7.03)		23.31 (5.77)		11.27 (3.79)		34.57 (8.62)	
Moderate	11.75 (2.86)		17.29 (4.43)		9.86 (3.13)		27.15 (6.60)		24.29 (5.81)		12.04 (3.85)		36.33 (8.71)	
High	12.73 (3.45)		18.76 (4.87)		10.72 (3.57)		29.48 (7.29)		23.82 (6.25)		11.41 (4.07)		35.23 (9.30)	
Self-rating popularity, *n* (%)	***	0.22	***	0.20	***	0.24	***	0.25		0.05	*	0.10		0.08
Less popular (≤ 7)	12.25 (3.11)		17.78 (4.72)		10.39 (3.32)		28.17 (6.96)		23.64 (5.94)		11.39 (3.92)		35.03 (8.87)	
Popular (> 7)	11.57 (3.15)		16.80 (4.80)		9.59 (3.34)		26.39 (7.15)		23.96 (5.94)		11.78 (3.89)		35.74 (8.88)	
**Parental mental health indicators**														
Father’s depression test, *n* (%)	***	0.33	***	0.22	***	0.25	***	0.26	***	0.26	***	0.21	***	0.26
Non-depression	11.61 (2.99)		17.08 (4.76)		9.87 (3.37)		26.95 (7.15)		24.22 (5.58)		11.85 (3.81)		36.07 (8.44)	
Depression	12.75 (3.50)		18.14 (4.78)		10.72 (3.61)		28.86 (7.28)		22.50 (6.60)		11.02 (4.00)		33.53 (9.72)	
Mother’s depression test, n (%)	***	0.35	***	0.17	***	0.23	***	0.23		0.10		0.08		0.10
Non-depression	11.54 (2.97)		16.91 (4.75)		9.68 (3.23)		26.59 (7.02)		24.05 (5.74)		11.81 (3.83)		35.86 (8.65)	
Depression	12.73 (3.38)		17.73 (4.75)		10.53 (3.62)		28.26 (7.23)		23.44 (6.02)		11.50 (3.91)		34.94 (9.11)	

***p < 0.001, **p < 0.1, *p < 0.05. Values that are presented in this table are means, standard deviations (SD), and effect sizes (Cohen’s d and partial eta square). Regarding effect size, we used Cohen’s d for independent two-sample t-tests and we used partial eta square ($\eta_{p}^{2}$) for one-way ANOVA. Effect sizes are indicated as small (0.2 ≤ d < 0.5, 0.01 ≤ $\eta_{p}^{2}$ < 0.06), medium (0.2 ≤ d < 0.5, 0.06 ≤ $\eta_{p}^{2}$ 0.14), and large(d ≥ 0.8,$\eta_{p}^{2}$ 0.14.

### 3.3. Associated factors for depression, problem behavior, and cognitive level

Table 3 presents the results of multivariate linear regression models regarding the associated factors for adolescent depression. Being a boy, having less than 8 h of sleep, fair/poor/very bad health status, low class ranking, high academic pressure, being less popular, and parental depression were significantly associated with a higher level of adolescent depression (*p*-values < 0.05).

**TABLE 3:** Multivariable linear regression analysis of the associated factors with depression in the imputed dataset (*n* = 2,463).

	Depression(*n* = 2,463)	
**Characteristic**	**β (95% CI)**	***P*-value**
**Demographic characteristic**		
Age	0.04 (−0.04, 0.11)	0.34
**Gender**		
Boys	Reference	
Girls	−0.26 (−0.50, −0.02)	0.03
**Birth order**		
First	Reference	
Second	−0.14 (−0.40, 0.12)	0.28
Third or more	0.34 (−0.12, 0.80)	0.24
**Mother’s education level**		
High school or higher	Reference	
Secondary school	0.30 (−0.11, 0.70)	0.16
Primary school or less	0.36 (−0.06, 0.78)	0.09
**Household income per year**		
4th quartile	Reference	
3rd quartile	0.11 (−0.22, 0.43)	0.53
2nd quartile	0.16 (−0.18, 0.50)	0.36
1st quartile	0.13 (−0.22, 0.48)	0.48
**Residence**		
Urban	Reference	
Rural	−0.07 (−0.33, 0.19)	0.59
**Children’s health-related factors**		
**Body mass index**		
Normal	Reference	
Underweight	0.13 (−0.14, 0.40)	0.33
Overweight/obesity	−0.03 (−0.37, 0.30)	0.85
**Sleeping duration**		
<8 h	Reference	
≥8 h	−0.40 (−0.69, −0.10)	0.01
**Self-rated health status**		
Excellent	Reference	
Good	0.42 (0.13, 0.71)	0.05
Fair	1.24 (0.94, 1.54)	<0.001
Poor and very bad	1.96 (1.35, 2.57)	<0.001
**School related factors**		
**Class ranking**		
0-10th percentile	Reference	
11th-75th percentile	0.21 (−0.08, 0.51)	0.16
76th-100th percentile	0.64 (0.20, 1.08)	0.01
**Academic pressure**		
Low	Reference	
Moderate	0.27 (−0.02, 0.55)	0.06
High	1.19 (0.89, 1.49)	<0.001
**Self-rated popularity**		
Popular (> 7)	Reference	
Less popular (≤ 7)	0.47 (0.23, 0.71)	<0.001
**Parental depression**		
**Father’s depression test**		
Non-depression	Reference	
Depression	1.16 (0.82, 1.51)	<0.001
**Mother’s depression test**		
Non-depression	Reference	
Depression	1.06 (0.75, 1.37)	<0.001
Rsquare (unadjusted)	0.13	
R square (adjusted)	0.12	

Table 4 shows the associated factors with adolescent problem behavior by multivariate linear regression analyses. Low education of mothers, poor or very bad health status, low class ranking, high academic pressure, being less popular at school, and parental depression were significantly associated with a higher score on the adolescent problem behavior test (*p*-values < 0.05). The patterns of associated factors regarding internalizing and externalizing problem behavior scores were similar to that of the total score.

**TABLE 4:** Multivariable linear regression analysis of the associated factors with problem behavior in the imputed dataset (*n* = 2,448).

Characteristics	Internalizing problem behavior		Externalizing problem behavior		Total Score	
	**β (95%CI)**	**P value**	**β (95%CI)**	**P value**	**β (95%CI)**	**P value**
**Demographic Characteristic**						
Age	0.12 (0.01, 0.23)	0.05	−0.041 (−0.12, 0.04)	0.31	0.08 (−0.09, 0.25)	0.38
**Gender**						
Boys	Reference		Reference		Reference	
Girls	−0.42 (−0.79, −0.05)	0.03	0.71 (0.45, 0.97)	<0.001	0.29 (−0.26, 0.84)	0.30
**Birth order**						
First	Reference		Reference		Reference	
Second	−0.30 (−0.71, 0.10)	0.14	−0.17 (−0.44, 0.56)	0.24	−0.47 (−1.07, 0.13)	0.12
Third or more	0.02 (−0.71, 0.74)	0.96	0.06 (−0.44, 0.56)	0.81	0.08 (−0.99, 1.14)	0.89
**Mother’s education level**						
High school or higher	Reference		Reference		Reference	
Secondary school	0.60 (−0.04, 1.23)	0.07	0.46 (0.02, 0.90)	0.04	1.06 (0.12, 1.99)	0.03
Primary school or less	1.26 (0.59, 1.93)	<0.001	1.09 (0.62, 1.55)	<0.001	2.35 (1.36, 3.34)	<0.001
**Household income per year**						
4th quartile	Reference		Reference		Reference	
3rd quartile	−0.26 (−0.78, 0.26)	0.33	0.06 (−0.31, 0.42)	0.76	−0.20 (−0.97, 0.57)	0.61
2nd quartile	−0.003 (−0.55, 0.54)	0.99	0.14 (−0.24, 0.52)	0.48	0.13 (−0.67, 0.94)	0.75
1st quartile	0.104 (−0.46, 0.66)	0.72	−0.08 (−0.47, 0.31)	0.68	0.02 (−0.80, 0.85)	0.96
**Residence**						
Urban	Reference		Reference		Reference	
Rural	−0.11 (−0.53, 0.31)	0.60	0.22 (−0.07, 0.51)	0.13	0.11 (−0.50, 0.72)	0.72
**Children’s health-related factors**						
**Body mass index**						
Normal	Reference		Reference		Reference	
Underweight	0.08 (−0.34, 0.50)	0.71	0.08 (−0.21, 0.38)	0.58	0.16 (−0.45, 0.78)	0.60
Overweight/obesity	0.36 (−0.17, 0.88)	0.18	0.33 (−0.03, 0.70)	0.08	0.69 (−0.08, 1.46)	0.08
**Sleeping duration**						
<8 h	Reference		Reference		Reference	
≥8 h	−0.02 (−0.48, 0.45)	0.95	0.24 (−0.09, 0.56)	0.15	0.22 (−0.46, 0.90)	0.52
**Self-rated health status**						
Excellent	Reference		Reference		Reference	
Good	0.40 (−0.05, 0.85)	0.08	0.03 (−0.28, 0.35)	0.83	0.43 (−0.23, 1.09)	0.20
Fair	1.14 (0.67, 1.61)	<0.001	0.64 (0.31, 0.97)	<0.001	1.78 (1.09, 2.47)	<0.001
Poor and very bad	1.29 (0.33, 2.25)	0.008	1.29 (0.62, 1.96)	<0.001	2.58 (1.17, 3.99)	<0.001
**School related factors**						
**Class ranking**						
0-10th percentile	Reference		Reference		Reference	
11-75th percentile	0.46 (−0.01, 0.93)	0.05	0.56 (0.24, 0.89)	0.01	1.03 (0.34, 1.71)	0.01
76th-100th percentile	0.23 (−0.46, 0.93)	0.51	1.04 (0.55, 1.52)	<0.001	1.27 (0.25, 2.29)	0.02
**Academic pressure**						
Low	Reference		Reference		Reference	
Moderate	0.99 (0.56, 1.43)	<0.001	0.33 (0.02, 0.63)	0.04	1.32 (0.68, 1.96)	<0.001
High	2.49 (2.02, 2.95)	<0.001	1.13 (0.81, 1.45)	<0.001	3.62 (2.93, 4.30)	<0.001
**Self-rated popularity**						
Popular (>7)	Reference		Reference		Reference	
Less popular (≤ 7)	0.81 (0.44, 1.19)	<0.001	0.66 (0.41, 0.92)	<0.001	1.48 (0.93, 2.02)	<0.001
**Parental depression**						
**Father’s depression test**						
Non-depression	Reference		Reference		Reference	
Depression	0.99 (0.45, 1.54)	<0.001	0.61 (0.23, 0.98)	0.01	1.60 (0.80, 2.40)	<0.001
**Mother’s depression test**						
Non-depression	Reference		Reference		Reference	
Depression	0.48 (−0.01, 0.97)	0.06	0.66 (0.32, 1.00)	<0.001	1.13 (0.42, 1.85)	0.01
R square (unadjusted)	0.09		0.11		0.11	
R square (adjusted)	0.08		0.10		0.10	

Table 5 demonstrates the results of associated factors for adolescents’ cognitive level by multivariate linear regression analyses. Younger age, being the third or higher birth order, low maternal education, low household income, living in a rural area, overweight/obesity, sleeping time less than 8 h per night, low class ranking, and father’s depression were significantly associated with adolescents’ cognitive level (*p*-values < 0.05). The patterns of associated factors regarding words and math tests were similar to that of the total score.

**TABLE 5:** Multivariable linear regression analysis of the associated factors with the cognitive level in the imputed dataset (*n* = 2106).

Characteristics	Word test		Math test		Total score	
	**β (95%CI)**	**P value**	**β (95%CI)**	**P value**	**β 95%CI)**	***P*-value**
**Demographic characteristic**						
Age	1.61 (1.48, 1.75)	<0.001	1.33 (1.25, 1.41)	<0.001	2.94 (2.76, 3.12)	<0.001
**Gender**						
Boys	Reference		Reference		Reference	
Girls	−0.45 (−0.88, −0.02)	0.04	0.04 (−0.21, 0.30)	0.74	−0.41 (−0.99, 0.18)	0.17
**Birth order**						
First	Reference		Reference		Reference	
Second	−0.37 (−0.84, 0.10)	0.12	0.07 (−0.22, 0.35)	0.64	−0.31 (−0.94, 0.33)	0.35
Third or more	−1.2 (−2.04, −0.37)	0.01	−0.33 (−0.83, 0.17)	0.20	−1.53 (−2.66, −0.40)	0.01
**Mother’s education level**						
High school or higher	Reference		Reference		Reference	
Secondary school	−0.74 (−1.50, 0.01)	0.05	−0.82 (−1.27, −0.27)	<0.001	−1.56 (−2.58, −0.54)	0.01
Primary school or less	−0.95 (−1.74, −0.17)	0.02	−1.09 (−1.56, −0.62)	< 0.001	−2.04 (−3.10, −0.98)	<0.001
**Household income per year**						
4th quartile	Reference		Reference		Reference	
3rd quartile	−0.48 (−1.08, 0.13)	0.12	−0.56 (−0.92, −0.21)	0.01	−0.73 (−1.38, −0.08)	0.01
2nd quartile	−0.52 (−1.14, 0.10)	0.10	−0.70 (−1.07, −0.33)	<0.001	−1.22 (−2.06, −0.39)	0.01
1st quartile	−0.87 (−1.50, −0.23)	0.01	−1.28 (−1.66, −0.90)	<0.001	−2.15 (−3.00, −1.29)	<0.001
**Residence**						
Urban	Reference		Reference		Reference	
Rural	−1.23 (−1.70,0.75)	<0.001	−0.65 (−0.93, 0.36)	<0.001	−1.87 (−2.52, 1.23)	<0.001
**Children’s health-related factors**						
**Body mass index**						
Normal	Reference		Reference		Reference	
Underweight	−0.32 (−0.81, 0.16)	0.20	−0.41 (−0.70, −0.12)	0.01	−0.73 (−1.38, −0.08)	0.03
Overweight/obesity	−0.54 (−1.15, 0.06)	0.08	−0.07 (−0.43, 0.29)	0.71	−0.61 (−1.43, 0.20)	0.14
**Sleeping duration**						
<8 h	Reference		Reference		Reference	
≥8 h	−0.50 (−1.04, 0.04)	0.07	−0.28 (−0.60,0.05)	0.10	−0.78 (−1.51, −0.044)	0.04
**Self-rated health status**						
Excellent	Reference		Reference		Reference	
Good	0.23 (−0.30, 0.75)	0.40	0.37 (0.06, 0.69)	0.02	0.60 (−0.11, 1.31)	0.10
Fair	0.28 (−0.27, 0.83)	0.32	−0.02 (−0.34, 0.31)	0.93	0.27 (−0.47, 1.00)	0.48
Poor and very bad	−0.99 (−2.08, 0.09)	0.07	0.23 (−0.42, 0.87)	0.50	−0.77 (−2.23, 0.69)	0.30
**School related factors**						
**Class ranking**						
0-10th percentile	Reference		Reference		Reference	
11-75th percentile	−1.05 (−1.60, −0.50)	<0.001	−0.53 (−0.86, −0.20)	0.01	−1.58 (−2.32, −0.84)	<0.001
76th-100th percentile	−3.58 (−4.49, −2.86)	< 0.001	2.39 (−2.88, −1.90)	<0.01	−6.06 (−7.17, −4.96)	<0.001
**Academic pressure**						
Low	Reference		Reference		Reference	
Moderate	0.15 (−0.36, 0.65)	0.57	0.12 (−0.18, 0.42)	0.44	0.27 (−0.42, 0.95)	0.45
High	−0.01 (−0.55, 0.53)	0.97	−2.3 9(−2.88, −1.90)	0.06	−0.33 (−1.06, 0.41)	0.38
**Self-rated popularity**						
Popular (>7)	Reference		Reference		Reference	
Less popular (≤ 7)	−0.06 (−0.49, 0.37)	0.79	−0.18 (−0.44, 0.07)	0.16	−0.24 (−0.83, 0.34)	0.41
**Parental depression**						
**Father’s depression test**						
Non-depression	Reference		Reference		Reference	
Depression	−1.61 (−2.24, −0.97)	< 0.001	−0.51 (−0.89, −0.13)	0.01	−2.12 (−2.97, −1.26)	<0.001
**Mother’s depression test**						
Non-depression	Reference		Reference		Reference	
Depression	−0.44 (−1.00, 0.12)	0.12	−0.27 (−0.60, 0.07)	0.12	−0.71 (−1.47, 0.05)	0.07
R square (unadjusted)	0.32		0.44		0.45	
R square (adjusted)	0.32		0.44		0.44	

Supplementary Tables 1–3 presents the coefficient betas, 95% confidence intervals, and *p*-values from the multilinear regression analysis based on the non-imputed data. The patterns of significant determinants were similar to those generated from the imputed datasets.

## 4. Discussion

In the present study, we examined an extensive set of determinants including demographic, socioeconomic, adolescent health-related, school- and family-related factors, in a national representative sample of 10–15 years old adolescents in China. The bivariate and multivariate analyses have mapped the different determinants of adolescent depression, problem behavior, and cognition. Among those determinants, some are modifiable, which can be used for developing more targeted public health policies, preventive strategies, and medical interventions.

To interpret results from the bivariate analyses, we should be aware that statistically significant differences do not necessarily indicate clinical relevance. In this study, we calculate effect sizes (i.e., Cohen’s d for independent two-sample *t*-tests and partial eta square ($\eta_{p}^{2}$) for one-way ANOVA). Effect sizes are indicated as small (0.2 ≤ d < 0.5 or 0.01 ≤ $\eta_{p}^{2}$ 0.06), medium (0.2 ≤ d < 0.5 or 0.06 ≤ $\eta_{p}^{2}$ 0.14), and large (d ≥ 0.8 or $\eta_{p}^{2}$ 0.14). Based on this classification criteria, large clinically significant differences were observed for the age of adolescents regarding word tests, math tests, and the total cognitive level. This finding was in line with the law of cognitive development, that is, the adolescent cognitive level generally increases with age (39). Despite age, most of the statistically significant differences in our study can be considered small differences in terms of their clinical relevance assessed by effect sizes. For instance, small clinical differences in adolescent depression were observed for the self-rated health status, self-rated popularity, academic pressure, father’s depression, and mother’s depression.

Multivariate analyses showed that several demographics, socioeconomic, adolescent health-related, and school- and family-related factors were associated with adolescent depression, problem behaviors, and cognition. Consistent with the bivariate analyses, some factors contributed statistically significantly but only slightly in the regression models. Despite all this, the present study provided detailed data on the pattern of determinants of adolescents’ mental health.

Our study found that being a boy, sleeping duration of fewer than 8 h, fair/poor/bad self-related health status, poor academic performance, high academic pressure, being less popular at school, and parents’ depression were associated with the relatively high level of adolescent’s self-rated depression. This finding from the regression analysis suggested that self-rated health, school-, and family-related factors may have greater independent impacts on the adolescent’s depression compared to demographic and socioeconomic factors. Depressed adolescents usually spent less time on their classes and homework, which may result in lower average grade points in examinations (12). They tend to have less optimal peer relationships, and fewer friends thus may become less popular at school (40). Also adolescents feel less connected to the classmates or friends and less popular at school may also be a key determinant of emotional depression (41). The strongest risk factors for adolescent depression are exposure to psychosocial stress and a family history of depression (42). Hence, our study highlighted the magnitudes of risk factors for adolescent depression especially the school and family-related factors that may consistently and closely expose adolescents to a psychologically distressing situation.

Regarding problem behaviors, we identified the risk factors including relatively low maternal education, fair/poor/bad self-reported health status, low-class ranking, moderate/high academic pressure, being less popular at school, and parents’ depression status. The patterns of risk factors for internalizing and externalizing problem behavior are similar to that for the total score of problem behavior. As reported by early studies, a low level of maternal education was a risk factor for children’s behavior problems, such as attention deficit hyperactivity disorder (ADHD) (43, 44). Children whose mothers had low education were more likely to endure adverse childhood experiences due to their mother’s lack of health literacy and lack of positive parenting skills, which may increase the risks of internalizing and externalizing behaviors (45). In China, academic performance strongly affects adolescents’ psychosocial well-being. Poor performance may impose the adolescent under negative evaluations by themselves and by peers, teachers, and family members. Our study also showed that parental depression was associated with the problem behavior of adolescents, and in particular, the association of a father’s depression with adolescent problem behavior was stronger than a mother’s depression, which was consistent with other reports (46). There is evidence of robust associations between fathers’ depressive affect and the problem behavior of their offspring (38, 47).

Our study found that the disadvantaged socioeconomic status indicated by low maternal education, low household income, and rural residence were related to lower cognitive levels of adolescents. One of the most well-established findings in developmental psychology is the strong relationship between maternal education and children’s health outcomes such as cognitive development (48, 49). Families with better socioeconomic status are capable to provide more resources for their children to get a higher quality of education, which is true worldwide. Adolescents who live in the rural area tend to have less educational resources since most of the good schools and well-educated teachers live in urban rather than the rural area which leads to lower cognitive levels of adolescents live in the rural. In addition, our study found a statistically significant association between a father’s depression and children’s cognitive level, while the association between a mother’s depression and children’s cognitive level was not statistically significant. Although we could not deny the importance of a mother’s psychological stress on children’s cognitive development, our study highlighted the important role of a father’s psychological status on children’s cognitive development. Evidence was rare regarding this issue. A study in the United Kingdom found that the influence of a father on children’s cognitive development may start even from a very young age (i.e., 3 months) (50).

Our study’s conclusions have significant clinical and public health implications regarding how to best serve China’s adolescent population’s mental health needs. It is critical to provide focused interventions and methods that address the risk variables found in this study given the significant prevalence of depression, problematic behaviors, and cognitive impairments among Chinese teenagers (47). Clinicians should be familiar with the identified risk factors and offer impacted adolescents assistance and interventions (28, 48, 49). Healthcare professionals, for instance, can screen teenagers for depression, problematic behaviors, and cognitive issues and offer early interventions to those who are at high risk (50). Teenagers and their families should also have access to appropriate mental health services and resources to ensure that they receive the proper support and care.

Our study has identified multiple risk factors for adolescents’ depression, problem behavior, and cognitive level. Based on the Cumulative Risk Model, a single risk factor cannot play a decisive role, but a comprehensive set of risk factors may have cumulative effects on adolescents’ mental health. Overall, our findings have clinical and public health implications, as some factors are modifiable and can be used for early interventions. Since family-related factors are important, an overall reduction of psychological distress in the family is recommended for future intervention. Mindfulness-based stress reduction and meditation may be recommended techniques to reduce families’ distress (51).

The Chinese Education Ministry released a policy named “double reduction” in 2021 in order to alleviate the burden of excessive homework and off-campus training among school-aged children and adolescents. In 2019, the Chinese National Health Commission published Healthy China Action Plan for Children and Adolescents Mental Health (2019–2022). This action plan aimed to build a society conducive to the mental health of children and adolescents by combining schools, communities, families, media, medical, and health institutions and implementing preventive interventions for psychological and behavioral problems and mental disorders of children and adolescents. The results of this study are useful for policymakers and public health specialists to make specialized public health policies and preventive interventions that address the modifiable risk factors.

### 4.1. Strengths and limitations

Overall, our efforts to investigate the determinants of depression, problem behavior and cognition in adolescents have extended the current literature by assessing a more comprehensive array of variables compared with previous studies and was conducted in such a nationally representative sample in China. This study was embedded into a population-based, prospective cross-sectional containing around 16,000 Chinese households in both urban and rural areas that were extracted from 25 provinces in China, which can represent 95% of the Chinese population. To our best knowledge, there may not be such a similar study in China. In addition, this study has valid measurements for both determinants and outcomes using reliable and standard instruments.

Even still, several limitations needed attention. First, although we aimed to include as many determinants as possible, there were still some associated factors that may not be measured and included in our study. This applies, for example, to early life stress, parent-adolescent interaction, and adverse experiences in early childhood (45, 47). Second, causation could not be evaluated due to the current cross-sectional analyses. The associations identified in our study may be in a binary direction. For instance, high academic pressure and being less popular at school may lead to the adolescent feeling depressed, or the other way around, a depressed individual may have poorer academic performance and lower popularity. Therefore, we called for caution when interpreting our results. Third, the depression of both adolescents and their parents was measured by a self-rated scale CES-D8 (48, 52). Although it was validated and widely used in epidemiological surveys, awareness is needed because it is not equal to the medical diagnosis (48). Fourth, as a longitudinal cohort, the CFPS study is not able to follow up with each participant for years. In 2012, 36% of the children were incrementally lost over the 6 years of follow-up (20). It is impossible to precisely compare the characteristics between people who were still in the study and those who were lost for follow-up. To deal with the issue of missing, we applied the multiple imputation approach, and we conducted multivariate regression analyses in both imputed and non-imputed datasets. The patterns of associated factors were similar in both datasets. Fifth, we included some subjective concepts in our study which may cause potential bias. However, this kind of bias might be alleviated by several approaches that have been already applied in the CFPS survey: CFPS group used the standard protocol or the standard operation procedure (SOP) to train the interviewers; questionnaires used for the survey were developed by experienced experts in relevant fields to make sure the question understandable, readable and concrete; and the present study involved a relatively large sample.

## 5. Conclusion

In this nationwide study, we have identified multiple associated factors, including the demographic, socioeconomic, health-related, and school-related factors of adolescents as well as their parent’s mental health indicators, for depression, problem behavior, and cognition among adolescents in China. These findings can provide insights into developing more targeted public health policies, preventive strategies, and medical interventions to improve adolescents’ mental health and promote their cognitive level. Support for adolescents is warranted by health policymakers, education authorities, health professionals, and families.

## Data availability statement

The original contributions presented in this study are included in the article/Supplementary material, further inquiries can be directed to the corresponding authors.

## Ethics statement

The studies involving human participants were reviewed and approved by the Ethics Committee of the Institution of Social Science Survey, Peking University (the approval number: IRB00001052-14010). Written informed consent to participate in this study was provided by the participants’ legal guardian/next of kin.

## Author contributions

YD, XH, GB, and LH were involved in the study conceptualization and study design. YD carried out statistical analyses. YD, XH, and GB wrote the first draft of the manuscript. LY, LS, JL, JX, YC, ZL, LH, and GB provided critical revisions of the article for important intellectual content. All authors contributed to the interpretation of the data and approved the final version of the article.
