# Supplementary material for: Determinants of depression, problem behavior, and cognitive level of adolescents in China: Findings from a national, population-based cross-sectional study
Source: Front Psychiatry. 2023 Apr 6;14:1159739. doi: 10.3389/fpsyt.2023.1159739 (PMC10119594; doi:10.3389/fpsyt.2023.1159739)
Supplement: Supplementary file 1 [file Table_1.docx]

Supplementary Material

Determinants of depression, problem behavior and cognitive level of adolescents in China: findings from a national, population-based cross-sectional study

Yusang Dong^1,†^, Xinyu He^2, †,^ Lizhen Ye^3^, Lidan Sun^1^, Jiabin Li^4^, Jingfang Xu^5^, Yuechong Cui^6^, Ziqiao Li^2^, Lidan Hu^1*^, Guannan Bai^2*^

* Correspondence:

Guannan Bai and Lidan Hu contributed as corresponding authors.

Correspondence to: guannanbai@zju.edu.cn; hulidan@zju.edu.cn

Supplementary Table S1. Multivariable linear regression analysis of the associated factors with depression in the non-imputed dataset (n=1731)

|  | Depression | | |
| --- | --- | --- | --- |
| Characteristics | β (95%CI) | *P*-value |  |
| Demographic Characteristic |  |  |  |
| Age | 0.01 (-0.10, 0.12) | 0.88 |  |
| Gender |  |  |  |
| Boys | Reference |  |  |
| Girls | -0.06 (-0.40, 0.28) | 0.73 |  |
| Birth order |  |  |  |
| First | Reference |  |  |
| Second | -0.37 (-0.74, -0.01) | 0.05 |  |
| Third or more | 0.25 (-0.47, 0.96) | 0.55 |  |
| Mother's education level |  |  |  |
| High school or higher | Reference |  |  |
| Secondary school | 0.33 (-0.17, 0.82) | 0.20 |  |
| Primary school or less | 0.60 (0.10, 1.10) | 0.02 |  |
| Household income per year |  |  |  |
| 4th quartile | Reference |  |  |
| 3rd quartile | 0.09 (-0.36, 0.55) | 0.69 |  |
| 2nd quartile | 0.10 (-0.58, 0.39) | 0.69 |  |
| 1st quartile | 0.16 (-0.37, 0.69) | 0.55 |  |
| Residence |  |  |  |
| Urban | Reference |  |  |
| Rural | 0.35 (-0.03, 0.72) | 0.07 |  |
| Children's health-related factors  fffafacfactors |  |  |  |
| Body Mass Index |  |  |  |
| Normal | Reference |  |  |
| Underweight | -0.01 (-0.38, 0.38) | 0.98 |  |
| Overweight/obesity | -0.31 (-0.78, 0.16) | 0.19 |  |
| Sleeping duration |  |  |  |
| <8 hours | Reference |  |  |
| ≥8 hours | -0.58 (-0.98, -0.17) | 0.01 |  |
| Self-rated health status |  |  |  |
| Excellent | Reference |  |  |
| Good | 0.23 (-0.18, 0.63) | 0.28 |  |
| Fair | 0.88 (0.45, 1.31) | <0.001 |  |
| Poor and very bad | 1.75 (0.68, 2.82) | 0.01 |  |
| School-related factors |  |  |  |
| Class ranking |  |  |  |
| 0-10th percentile | Reference |  |  |
| 11-75th percentile | 0.48 (0.09, 0.87) | 0.02 |  |
| 76th -100th percentile | 1.08 (0.35, 1.81) | 0.01 |  |
| Academic pressure |  |  |  |
| Low | Reference |  |  |
| Moderate | 0.42 (0.03, 0.82) | 0.04 |  |
| High | 1.38 (0.95, 1.81) | <0.001 |  |
| Self-rated popularity |  |  |  |
| Popular (>7) | Reference |  |  |
| Less popular (≤7) | 0.48 (0.14, 0.82) | 0.01 |  |
| Parental depression |  |  |  |
| Father's depression test |  |  |  |
| Non-depression | Reference |  |  |
| Depression | 0.83 (0.38, 1.29) | <0.001 |  |
| Mother's depression test |  |  |  |
| Non-depression | Reference |  |  |
| Depression | 0.73 (0.32, 1.13) | 0.001 |  |
| R square(unadjusted) | 0.14 |  |  |
| R square(adjusted) | 0.12 |  |  |

Supplementary Table S2. Multivariable linear regression analysis of the associated factors with problem behavior in the non-imputed dataset (n=1763)

| Characteristics | Internalizing problem behavior | | Externalizing problem behavior | | Total score | |
| --- | --- | --- | --- | --- | --- | --- |
|  | β (95%CI) | *P*-value | β (95%CI) | *P*-value | β (95%CI) | *P*-value |
| Demographic Characteristic |  |  |  |  |  |  |
| Age | 0.23 (0.07, 0.40) | 0.01 | 0.04 (-0.08, 0.15) | 0.53 | 0.27 (0.03, 0.51) | 0.03 |
| Gender |  |  |  |  |  |  |
| Boys | Reference |  | Reference |  | Reference |  |
| Girls | -0.05 (-0.60, 0.49) | 0.85 | 0.91 (0.52, 1.29) | <0.001 | 0.85 (0.04, 1.66) | <0.001 |
| Birth order |  |  |  |  |  |  |
| First | Reference |  | Reference |  | Reference |  |
| Second | -0.44 (-1.04, 0.16) | 0.15 | -0.23 (-0.65, 0.20) | 0.30 | -0.67 (-1.56, 0.23) | 0.15 |
| Third or more | -0.24 (-1.40, 0.93) | 0.69 | 0.38 (-0.44, 1.19) | 0.37 | 0.14 (-1.59, 1.87) | 0.87 |
| Mother's education level |  |  |  |  |  |  |
| High school or higher | Reference |  | Reference |  | Reference |  |
| Secondary school | 0.36 (-0.48, 1.20) | 0.40 | 0.41 (-0.18, 1.01) | 0.17 | 0.75 (-0.50, 2.00) | 0.24 |
| Primary school or less | 1.08 (0.18, 1.97) | 0.02 | 0.96 (0.33, 1.59) | 0.01 | 2.01 (0.69, 3.34) | 0.01 |
| Household income per year |  |  |  |  |  |  |
| 4th quartile | Reference |  | Reference |  | Reference |  |
| 3rd quartile | -0.11 (-0.85, 0.63) | 0.77 | -0.01 (-0.52, 0.52) | 0.99 | -0.11(-1.21, 0.98) | 0.84 |
| 2nd quartile | 0.34 (-0.44, 1.12) | 0.39 | 0.02 (-0.53, 0.57) | 0.95 | 0.36 (-0.80, 1.52) | 0.54 |
| 1st quartile | 0.29 (-0.57, 1.15) | 0.51 | -0.02 (-0.63, 0.59) | 0.94 | 0.27 (-1.01, 1.55) | 0.68 |
| Residence |  |  |  |  |  |  |
| Urban | Reference |  | Reference |  | Reference |  |
| Rural | 0.36 (-0.24, 0.96) | 0.24 | -0.20 (-0.61, 0.23) | 0.38 | 0.17 (-0.72, 1.07) | 0.71 |
| Children's health-related factors |  |  |  |  |  |  |
| Body Mass Index |  |  |  |  |  |  |
| Normal | Reference |  | Reference |  | Reference |  |
| Underweight | 0.10 (-0.52, 0.72) | 0.75 | 0.22 (-0.22, 0.65) | 0.33 | 0.22 (-0.22, 0.65) | 0.33 |
| Overweight/obesity | 0.52 (-0.25, 1.28) | 0.19 | 0.33 (-0.21, 0.86) | 0.24 | 0.33 (-0.21, 0.86) | 0.24 |
| Sleeping duration |  |  |  |  |  |  |
| <8 hours | Reference |  | Reference |  | Reference |  |
| ≥8 hours | -0.30 (-0.96, 0.36) | 0.38 | -0.03 (-0.50, 0.44) | 0.90 | -0.03 (-0.50, 0.44) | 0.90 |
| Self-rated health status |  |  |  |  |  |  |
| Excellent | Reference |  | Reference |  | Reference |  |
| Good | 0.34 (-0.31, 0.99) | 0.31 | 0.16 (-0.30, 0.62) | 0.50 | 2.53 (-0.05, 5.11) | 0.55 |
| Fair | 0.95 (0.25, 1.64) | 0.01 | 0.65 (0.16, 1.14) | 0.01 | 1.60 (0.57, 2.64) | 0.01 |
| Poor and very bad | 1.49 (-0.24, 3.22) | 0.10 | 1.04 (-0.18, 2.26) | 0.10 | 0.50 (-0.48, 1.48) | 0.03 |
| School-related factors |  |  |  |  |  |  |
| Class ranking |  |  |  |  |  |  |
| 0-10th percentile | Reference |  | Reference |  | Reference |  |
| 11-75th percentile | 0.89 (0.25, 1.54) | 0.01 | 1.15 (0.71, 1.59) | <0.001 | 2.04 (1.10, 2.98) | <0.001 |
| 76th-100th percentile | 2.68 (1.98, 3.37) | 0.01 | 2.31 (1.46, 3.14) | <0.001 | 4.07 (2.30, 5.84) | <0.001 |
| Academic pressure |  |  |  |  |  |  |
| Low | Reference |  | Reference |  | Reference |  |
| Moderate | 0.89 (0.25, 1.53) | 0.01 | 0.30 (-0.15, 0.75) | 0.20 | 1.20 (0.24, 2.16) | 0.02 |
| High | 2.67 (1.98, 3.37) | 0.01 | 0.96 (0.47, 1.45) | <0.001 | 3.64 (2.60, 4.67) | <0.001 |
| Self-rated popularity |  |  |  |  |  |  |
| Popular (>7) | Reference |  | Reference |  | Reference |  |
| Less popular (≤7) | -0.43 (-0.98, 0.11) | 0.12 | 0.77 (-0.38, 1.15) | <0.001 | -1.20 (-2.00, -0.38) | <0.001 |
| Parental depression |  |  |  |  |  |  |
| Father's depression test |  |  |  |  |  |  |
| Non-depression | Reference |  | Reference |  | Reference |  |
| Depression | 0.96 (0.23, 1.70) | 0.01 | 0.45 (-0.08, 0.96) | 0.09 | 1.40 (0.31, 2.50) | 0.01 |
| Mother's depression test |  |  |  |  |  |  |
| Non-depression | Reference |  | Reference |  | Reference |  |
| Depression | 0.55 (-0.11, 1.22） | 0.10 | 0.66 (0.19, 1.12) | 0.01 | 1.21 (0.22, 2.20) | 0.02 |
| R square(unadjusted) | 0.12 |  | 0.14 |  | 0.15 |  |
| R square(adjusted) | 0.10 |  | 0.13 |  | 0.13 |  |

Supplementary Table S3. Multivariable linear regression analysis of the associated factors with the cognitive level in the non-imputed dataset (n=1714)

| Characteristics | Word test | | Math test | | Total score | |
| --- | --- | --- | --- | --- | --- | --- |
|  | β (95%CI) | *P*-value | β (95%CI) | *P*-value | β (95%CI) | *P*-value |
| Demographic Characteristic |  |  |  |  |  |  |
| Age | 1.61 (1.42,1.79) | <0.001 | 1.36(1.24,1.48) | <0.001 | 2.97（2.71,3.22） | <0.001 |
| Gender |  |  |  |  |  |  |
| Boys | Reference |  | Reference |  | Reference |  |
| Girls | -0.41 (-0.99,0.18) | 0.17 | 0.22 (-0.17, 0.61) | 0.28 | -0.18 (-0.99, 0.62) | 0.65 |
| Birth order |  |  |  |  |  |  |
| First | Reference |  | Reference |  | Reference |  |
| Second | -0.21 (-0.80,0.48) | 0.52 | -0.22 (-0.64,0.21) | 0.32 | -0.42 (-1.30,0.47) | 0.36 |
| Third or more | -2.15 (-3.39, -0.92) | 0.01 | -0.86 (-1.69, -0.03) | 0.04 | -3.00 (-4.72, -1.29) | 0.01 |
| Mother's education level |  |  |  |  |  |  |
| High school or higher | Reference |  | Reference |  | Reference |  |
| Secondary school | -1.16 (-2.06, -0.27) | 0.01 | 0.66 (-1.26, -0.06) | 0.03 | -1.84 (-3.08, -0.60) | 0.01 |
| Primary school or less | -1.74 (-2.69, -0.78) | <0.001 | -1.40 (-2.04, -0.76) | <0.001 | -3.11 (-4.44, -1.79) | <0.001 |
| Household income per year |  |  |  |  |  |  |
| 4th quartile | Reference |  | Reference |  | Reference |  |
| 3rd quartile | -0.03 (-0.82,0.77) | 0.95 | -0.28 (-0.80, 0.25) | 0.31 | -0.31 (-1.41,0.79) | 0.57 |
| 2nd quartile | 0.31 (-0.51,1.13) | 0.57 | -0.52 (-1.07,0.03) | 0.06 | -0.22 (-1.36,0.92) | 0.70 |
| 1st quartile | -0.67 (-1.58,0.24) | 0.15 | -1.00 (-1.61, -0.40) | 0.01 | -1.67 (-2.93, -0.41) | 0.01 |
| Residence |  |  |  |  |  |  |
| Urban | Reference |  | Reference |  | Reference |  |
| Rural | -0.32 (-0.96, 0.32) | 0.36 | -0.39 (-0.03, 082) | 0.07 | -0.71 (-1.59, 0.17) | 0.12 |
| Children's health-related factors |  |  |  |  |  |  |
| Body Mass Index |  |  |  |  |  |  |
| Normal | Reference |  | Reference |  | Reference |  |
| Underweight | -0.36(-1.02,0.29) | 0.27 | -0.30(-0.73,0.14) | 0.18 | -0.73(-1.63,0.18) | 0.12 |
| Overweight/obesity | -0.66(-1.46,0.15) | 0.11 | -0.22(-0.76,0.31) | 0.32 | -0.90(-2.02,0.23) | 0.12 |
| Sleeping duration |  |  |  |  |  |  |
| <8 hours | Reference |  | Reference |  | Reference |  |
| ≥8 hours | 0.52(-0.10, 1.12） | 0.10 | -0.38(-0.84,0.10) | 0.12 | -0.97 (-1.94,0.01) | 0.05 |
| Self-rated health status |  |  |  |  |  |  |
| Excellent | Reference |  | Reference |  | Reference |  |
| Good | 0.08 (-0.60,0.76) | 0.81 | 0.18(-0.29,0.64) | 0.45 | -0.87 (-3.31,1.57） | 0.49 |
| Fair | 0.34 (-0.38,0.03） | 0.25 | -0.05(-0.54,0.45) | 0.85 | 0.30 (-0.73,1.33) | 0.58 |
| Poor and very bad | -1.04 (-2.62,0.90) | 0.34 | 0.35(-0.99,1.35) | 0.77 | 0.17(-0.80, 1.14) | 0.73 |
| School-related factors |  |  |  |  |  |  |
| Class ranking |  |  |  |  |  |  |
| 0-10th percentile | Reference |  | Reference |  | Reference |  |
| 11-75th percentile | -0.72 (-1.39, -0.04） | 0.04 | -0.35 (-0.80, 0.11) | 0.14 | -1.00(-2.02, -0.13) | 0.03 |
| 76th -100th percentile | -4.13 (-5.38, -2.89) | <0.001 | -1.79 (-2.62, -0.96) | <0.001 | -5.58(-7.30, -3.85) | <0.001 |
| Academic pressure |  |  |  |  |  |  |
| Low | Reference |  | Reference |  | Reference |  |
| Moderate | 0.56 (-0.12, 1.24) | 0.11 | 0.23 (-0.23, 0.68) | 0.32 | 0.62 (-0.31, 1.59） | 0.17 |
| High | 0.38 (-0.36, 1.12） | 0.32 | -0.34 (-0.84, 0.15) | 0.17 | 0.07 (-0.96, 1.10） | 0.90 |
| Self-rated popularity |  |  |  |  |  |  |
| Popular (>7) | Reference |  | Reference |  | Reference |  |
| Less popular (≤7) | -0.23 (-0.81, 0.35) | 0.45 | -0.20 (-0.59, 0.19） | 0.32 | -0.40 (-1.20, 0.41） | 0.33 |
| Parental depression |  |  |  |  |  |  |
| Father's depression test |  |  |  |  |  |  |
| Non-depression | Reference |  | Reference |  | Reference |  |
| Depression | -0.54 (-1.33,0.25） | 0.17 | -0.11(-0.64,0.43) | 0.70 | -0.64 (-1.74, 0.45) | 0.25 |
| Mother's depression test |  |  |  |  |  |  |
| Non-depression | Reference |  | Reference |  | Reference |  |
| Depression | 0.24 (-0.47,0.94） | 0.51 | -0.05(-0.52,0.43） | 0.85 | 0.19 (-0.79, 1.17) | 0.70 |
| R square(unadjusted) | 0.35 |  | 0.45 |  | 0.48 |  |
| R square(adjusted) | 0.34 |  | 0.43 |  | 0.47 |  |
